# Rolling behavior of a micro-cylinder in adhesional contact

**DOI:** 10.1038/srep34063

**Published:** 2016-09-28

**Authors:** Shigeki Saito, Toshihiro Ochiai, Fumikazu Yoshizawa, Ming Dao

**Affiliations:** 1 Dept. of Mechanical and Aerospace Engineering, Tokyo Institute of Technology, 2-12-1 O-okayama, Meguro-ku, Tokyo, 152-8552, Japan; 2 Dept. of Materials Science and Engineering, Massachusetts Institute of Technology, 77 Massachusetts Avenue, Cambridge, MA 02139, USA

## Abstract

Understanding the rolling behavior of a micro-object is essential to establish the techniques of micro-manipulation and micro-assembly by mechanical means. Using a combined theoretical/computational approach, we studied the critical conditions of rolling resistance of an elastic cylindrical micro-object in adhesional contact with a rigid surface. Closed-form dimensionless expressions for the critical rolling moment, the initial rolling contact area, and the initial rolling angle were extracted after a systematic parametric study using finite element method (FEM) simulations. The total energy of this system is defined as the sum of three terms: the elastic energy stored in the deformed micro-cylinder, the interfacial energy within the contact area, and the mechanical potential energy that depends on the external moment applied to the cylindrical micro-object. A careful examination of the energy balance of the system surprisingly revealed that the rolling resistance per unit cylindrical length can be simply expressed by “*work of adhesion times cylindrical radius*” independent of the Young’s modulus. In addition, extending a linear elastic fracture mechanics based approach in the literature, we obtained the exact closed-form asymptotic solutions for the critical conditions for initial rolling; these asymptotic solutions were found in excellent agreement with the full-field FEM results.

Many of the micro-scale mechanical systems such as micro-electro-mechanical systems (MEMS) have been developed in recent years. Rapid improvements in the manufacturing process of silicon chips enabled such trends. The demand for producing highly integrated functions in ever smaller dimensions significantly increases, which we can see obviously in present-day industrial products. Although the current techniques based on the photolithography, which are often used in manufacturing integrated circuits, enable us to produce layered structures that work as MEMS, in order to realize highly functional systems, precise manipulation and assembly of micro-parts into a fully three dimensional structure is recognized to be still indispensable. Additionally, the techniques of micro-manipulation and assembly could be helpful to investigate scientific phenomena in micro/nano world. For instance, the development of intelligent materials that can play roles as photonic band devices was reported^1^. As described in the article^1^, the micro-manipulation technique includes three processes: picking a micro-object from a substrate, carrying and then placing it to an arbitrary point. By the repetition of these processes, a three dimensional structure can be constructed. In such processes, the use of gripper mechanism is effective down to the millimeter scale. In the micrometer (or even smaller) scale, however, it is not effective anymore to execute these processes by the gripper mechanism because the adhesional force that causes the micro-object to unexpectedly adhere to one of the gripper fingers becomes much greater than the gravitational force due to the scale effect. Therefore, it becomes difficult to achieve high precision and repeatability by the gripper mechanism in micro-manipulation^2^.

In order to realize the technique of micro-manipulation with high precision and repeatability, a mechanical technique of micro-manipulation by a single probe was suggested^3^,^4^. This technique considers the change of mechanical interaction versus the force variation from the probe-tip. Because the probe-object or object-substrate interface could be selectively broken with the use of the technique, picking-up and placing objects can be more repeatable and precisely controlled. In parallel, it is critical to understand the contact mechanics of an elastic particle in adhesional contact with a substrate. For example, the Johnson-Kendall-Roberts (JKR) model^5^ of elastic contact under a compressive load has found tremendous applications in micromanipulation^6^ (including adhesional contact of biological cells^7^).

Although the rolling-resistance of a micro-object was mentioned in the literature^3^,^4^, a quantitative understanding is still missing and, consequently, the high repeatability required during micro-manipulation cannot be achieved. Recently, some researchers have tried to evaluate the order of magnitude of the rolling-resistance via their theoretical and/or experimental approaches^8^,^9^,^10^,^11^,^12^. For example, Soltani and Ahmadi^8^ theoretically discussed the rolling of a spherical and a cylindrical object in terms of geometry and rotational moment around a certain axis, but did not provide a reasonable criterion for the critical point of rolling. Dominik and Tielens^12^, for the first time as far as we know, explicitly mentioned the existence of rolling resistance beyond which an irreversible rolling of an object occurs and analytically calculated the relation between the moment opposing the attempt to roll and angular displacement; however, they did not clarify which factors determine the critical status from not-rolling to rolling. She *et al.*^11^ reported the relation of the adhesion hysteresis and rolling contact mechanics via theoretical and experimental approaches using polymer materials in terms of crack propagation along the edges of the contact interface; still they did not reveal the transition mechanism from quasi-static status to dynamic status (mentioned as “instability”). Peri and Cetinkaya^10^ and Ding *et al.*^9^ experimentally demonstrated the existence of a critical rolling resistance of a microsphere. For interpreting the experimental results, they adopted Dominik and Tielens’ theory which did not explain the dominant factors of the critical condition. Barquins *et al.*^13^, She *et al.*^11^, and Greenwood *et al.*^14^ established a linear elastic fracture mechanics (LEFM) based asymptotic method to study the functional dependences for the problem of rolling; nevertheless, in these earlier studies, explicit expressions for the critical conditions of initial rolling were not fully developed. To date, a model sufficiently convincing and theoretically consistent for explaining the critical status of the rolling behavior in micro-scale is not yet available.

To solve nonlinear contact mechanics problems where exact closed-form solutions are rather difficult to obtain, it has been proven practically very useful to derive approximate closed-form dimensionless functions from parametric computational simulations^15^,^16^. Therefore, in this study, we calculate rolling resistances of micro-cylinders for a few kinds of materials in terms of the total energy and extract the normalized dimensionless functions in order to understand the rolling behavior of a micro-object in adhesional contact. To simplify the problem, we assume an isotropic and elastic cylindrical micro-object in adhesional contact with a rigid surface. The plane strain condition is assumed. Once an external moment is applied, we can calculate the total energy of this system consisting of the following three terms: the elastic energy stored in the deformed micro-cylinder, the interface energy within the contact area, and the mechanical potential energy that depends on the external moment. Then we adopt the finite element method (FEM) to compute the elastic energy. The total energy is obtained as the function of contact area. We identify the equilibrium state of the system by finding the local minimum value of the total energy. We determine the rolling resistance for various conditions by obtaining the critical external moment at which the micro-cylinder makes the transition from equilibrium static state to non-equilibrium rolling state. The normalized dimensionless functions of rolling resistance can be deduced by plotting numerical solutions for various parameters in logarithmic scales. In addition, extending the asymptotic LEFM approach developed in the literature^11^,^13^,^14^, explicit critical rolling resistant conditions can be obtained. Direct comparison and cross-validation are given between the asymptotic LEFM based analytical solutions and the full-field FEM/dimensional analysis based results.

## Results

### Theoretical considerations

**Figure 1:**
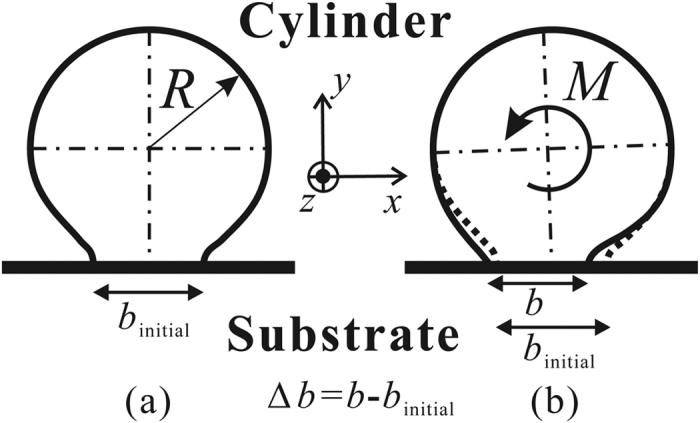
Cross-sectional schematic illustration of the rolling process of an elastic micro-cylinder adhered to a rigid substrate. (**a**) The initial state, and (**b**) propagation of the contact width under an external moment *M*.

Figure 1 shows the schematic illustration of the rolling process of an elastic micro-cylinder, which we consider in this study. Assuming no strain in the *z* direction, the adhesional contact between the substrate and the micro-cylinder is treated as a plane strain problem. The rolling process is taken to be quasi-static. Figure 1(a) shows the initial state that is the adhesional contact between the substrate and the micro-cylinder with neither moment nor force applied. As shown in Fig. 1(a), *R* represents the radius of the micro-cylinder and *b*_initial_ represents the initial width of the contact area without any applied moment or force. According to the theory proposed by Kalker^17^ and Barquins^13^, *b*_inital_ is given by


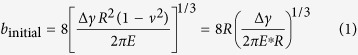


where Δ*γ* is the work of adhesion of the interface between the cylinder and the substrate, *ν* is the Poisson’s ratio, *E* is the Young’s modulus, and 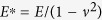 is the reduced modulus. The substrate is taken to be a rigid flat surface, and the micro-cylinder to be linearly elastic and isotropic. We assume no friction between the substrate and the micro-cylinder.

Figure 1(b) shows the loaded state, when an external moment *M* is applied to the center of the cylindrical micro-object. Consequently, the width of the adhesional contact area between the substrate and the cylindrical micro-object reduces and varies from *b*_initial_ to *b*. As shown in Fig. 1(b), Δ*b* is the variation in the width of the contact area from *b*_initial_ to *b*. Therefore, Δ*b* can be given as


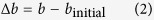


Since the positive direction of *b* is defined as the incremental direction of *b*, Δ*b* is negative. The change in the width of the contact area represents the propagation of the adhesional contact surface between the substrate and the cylindrical micro-object. When *U*_total_ is defined as the total energy of this system with a given *M*, *U*_total_ varies with *b*. In other words, *U*_total_ can be computed once *M* and *b* are both defined.

**Figure 2:**
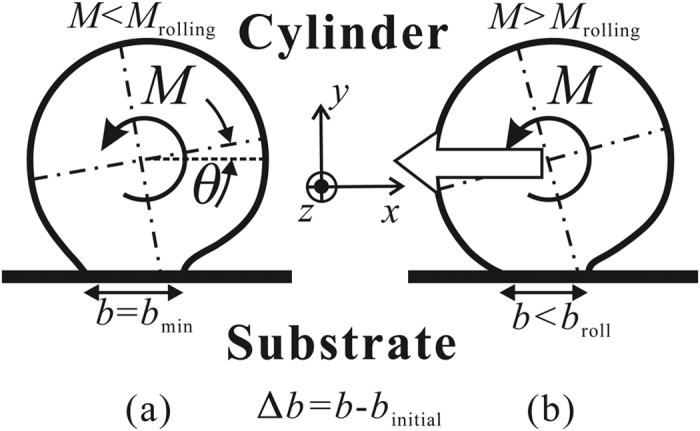
Cross-sectional schematic illustration of the energy state of the cylindrical micro-object under an applied moment *M*. (**a**) The equilibrium state, and (**b**) the state when the cylindrical micro-object begins to roll.

**Figure 3:**
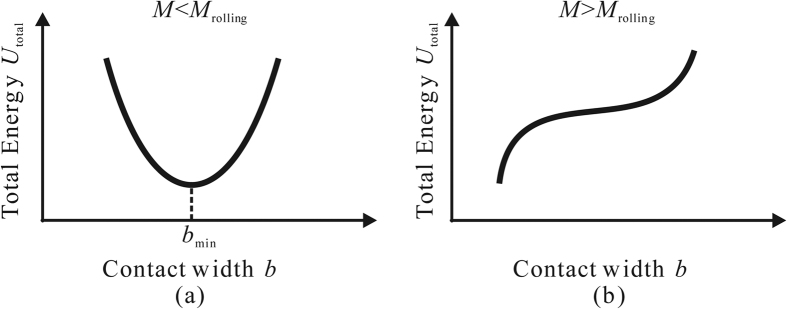
Schematic illustration of the relation between total energy *U*_total,_ and contact width *b* under an external moment *M*. (**a**) The equilibrium state, and (**b**) the state when the cylindrical micro-object begins to roll.

**Figure 4:**
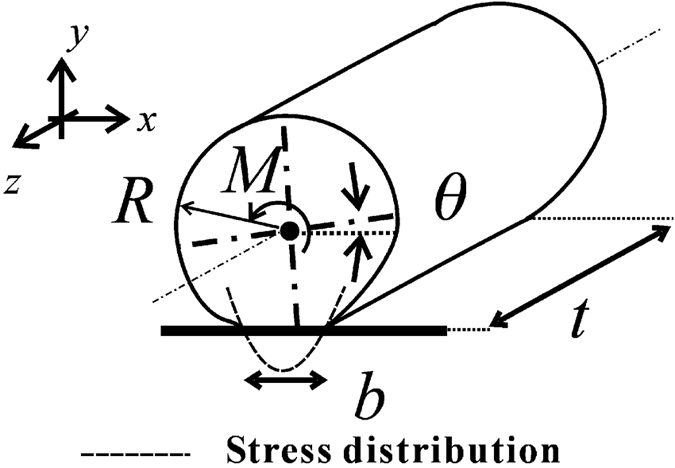
Isometric schematic illustration of the model of the micro-cylinder for calculating total energy *U*_total_, rotational angle *θ*, and contact width *b* for an external moment *M* given.

The rolling resistance of the micro-cylinder, *M*_roll_, is defined as the external moment that enables the cylindrical micro-object to start rolling. As illustrated in Fig. 2(a), if the external moment *M* is smaller than *M*_roll_, the micro-cylinder is in equilibrium with the given external moment *M*, and adheres to the substrate stably. In Fig. 2(a), *b*_min_ and *θ* represent the width of the contact area and the deformed angle in the equilibrium state respectively. In this case, among all possible elasticity solutions with a contact width *b*, *b*_min_ minimizes *U*_total_, as shown in Fig. 3(a). In other words, if the adhesional contact surface propagates quasi-statically along the interface between the micro-cylinder and the substrate, the contact width *b* reaches its minimum, *b*_min_, at the equilibrium state. However, if the given moment *M* is greater than *M*_roll_, *b*_min_ does not exist and the micro-cylinder begins to roll as illustrated in Figs 2(b) and 3(b). In summary, the state of the micro-cylinder with *M* applied depends on the balance between *U*_total_ and *b* with *M* given. The total energy of this system, *U*_total_, is given as,


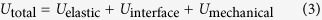


where *U*_elastic_ is the elastic energy that is stored in the cylindrical micro-object, *U*_interface_ is the interfacial energy that is stored in the interface between the substrate and the micro-cylinder, and *U*_mechanical_ is the potential energy that is defined with the external moment *M*. *U*_interface_ and *U*_mechanical_ are given by the following equations respectively:


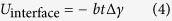



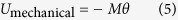


where *t* is the thickness, i.e., the length of the cylindrical micro-object in the *z* direction (see Fig. 4).

In order to calculate the total energy *U*_total_, the rotation angle *θ*, and the contact width *b* for a given external moment *M*, systematic simulations were conducted using FEM simulations. In order to obtain the elastic energy *U*_elastic_ with a contact width *b*, the constraint condition is given to the contact interface, and the external moment *M* is applied to the axis of the micro-cylinder. As a result, the stress is distributed along the interface due to the adhesional force as illustrated in Fig. 4 by the broken line. The right-hand-side edge (trailing edge) of the contact area behaves similarly as the crack front where the resultant stress is singular. The left-hand-edge (leading edge) however behaves differently, where no stress singularity exists and essentially zero stress is found along the leading edge. This means no new interface is formed due to the external moment *M* as long as the quasi-static loading and JKR potential for interface are assumed. The value of *U*_total_ is numerically obtained as a function of *b*. While the stress states on both edges are carefully monitored in the FEM simulations, it is noted that the criterion of reaching the critical conditions for incipient (initial) rolling is governed by the total energy *U*_total_ (see also Fig. 3) instead of the stress state (or stress singularity) on the edges.

**Figure 5:**
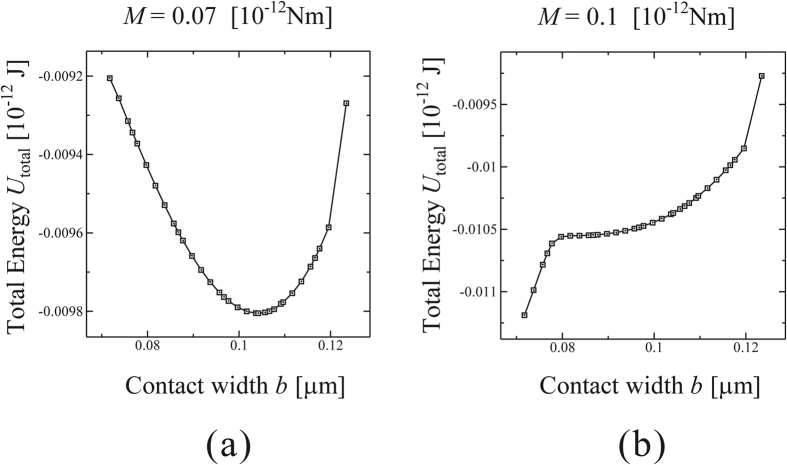
Relation between total energy *U*_total_ and contact width *b*. (**a**)The equilibrium state for *M* = 0.07 [10^−12^ Nm], and (**b**) the state when the micro-cylinder begins to roll for *M* = 0.1 [10^−12^ Nm].

We used the commercially available FEM package ABAQUS Standard (SIMULIA, Providence, RI, USA) for numerical simulations. As an example, the elastic energies of the micro-cylinder with different values of contact width *b* were calculated with respect to various external moments given. In the first example studied, the radius and thickness of the micro-cylinder are both 1 [μm]. The cylinder material assumed in the model is polystyrene, where Young’s modulus *E* = 3.8 [GPa], and Poisson’s ratio *ν* = 0.34, and the work of adhesion between the cylinder and the substrate is taken as Δ*γ* = 0.1 [N/m]^3^. Figure 5(a) shows the relation between total energy *U*_total_ and contact width *b* for an external moment *M* = 0.07 [10^−12^ Nm] given. As shown in Fig. 5(a), *U*_total_ reaches the local minimum value for *b* = 0.104 [μm]. Therefore, at *M* = 0.07 [10^−12^ Nm], the micro-cylinder is in equilibrium with the external moment for *b* = 0.104 [μm].

**Figure 6:**
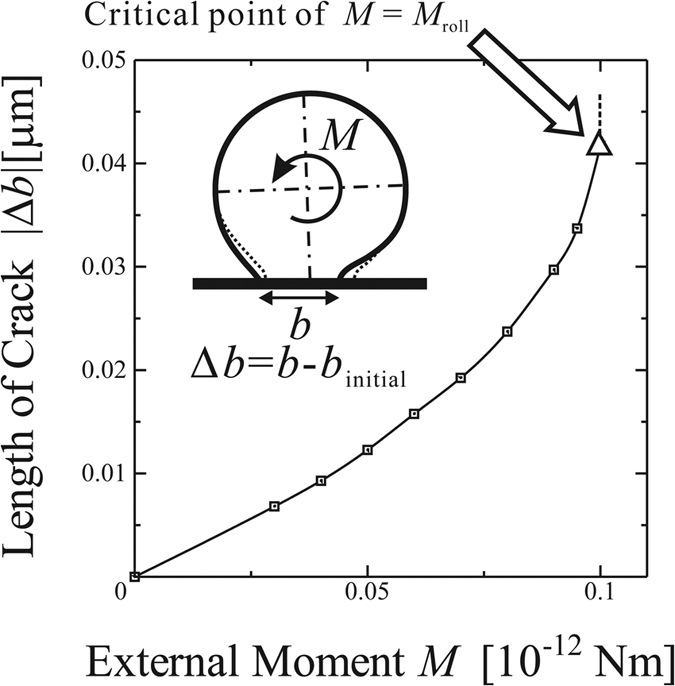
Relation between the external moment, *M*, and the reduction of the contact length, |Δ*b*|, equilibrated with the corresponding external moment. The point for *M* = 0.1 [10^−12^ Nm] is the critical point of *M* = *M*_roll_. Beyond and at this point, no *b*_min_ is found and no equilibrium is possible. See text for more details.

Figure 5(b) shows the relation between total energy *U*_total_ and contact width *b* for an external moment *M* = 0.1 [10^−12^ Nm] given. As demonstrated in Fig. 5(b) and discussed earlier with Fig. 3(b), the local minimum point does not exist anymore. Therefore, at *M* = 0.1 [10^−12^ Nm], the micro-cylinder can not be in equilibrium with the external moment any more, and has to roll. In the case of *M* = 0.105 and 0.11 [10^−12^ Nm], no minimum *U*_total_ can be found. Figure 6 shows the relation between the reduction of the contact length, |Δ*b*|, and the corresponding external moment *M*. The critical value of the rolling moment, *M*_roll_, is approximately 0.1 [10^−12^ Nm]. The error of *M*_roll_ ranges within ±5% of the estimated value 0.1 [10^−12^ Nm], because the equilibrium state can be definitely confirmed with *M* = 0.095 [10^−12^ Nm], but not with *M* = 0.105 [10^−12^ Nm] anymore. Although the static rolling resistance between a pair of friction-free solid surfaces seems to be zero in the macro scale, such rolling resistance has a certain finite value in micro scale due to adhesional effect. The critical rolling conditions can be determined by evaluating the balance of the total energy, *U*_total_, which is influenced by both the external moment and the adhesional effect between the substrate and the micro-cylinder.

### General dimensionless functions for micro-cylinder adhesional rolling

**Figure 7:**
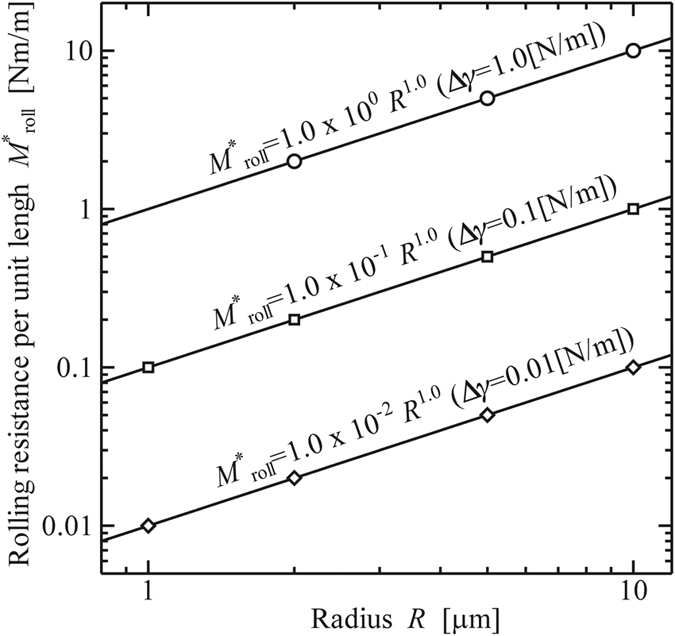
Rolling resistance per unit length at the beginning of rolling, 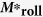 [10^−6^ Nm/m]. (Material: polystyrene, *E* = 3.8 [GPa], *ν* = 0.34)**.**

For various radii *R* of a polystyrene micro-cylinder (*E* = 3.8 [GPa], *ν* = 0.34) and interfacial works of adhesion Δ*γ*, we calculated the rolling resistance per unit length, 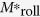 [10^−6^ Nm/m], the corresponding width of contact area at the beginning of rolling, *b*_roll_, and the rotational angle at the beginning of rolling, *θ*_roll_, where the errors are all kept within ±5% in each case. Figure 7 shows the relation between 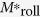 and *R* for Δ*γ* = 0.01, 0.1, and 1.0 [N/m]. Each solid line is obtained by fitting a line to the plotted data with the least-square method. The results clearly show that 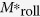 is proportional to the radius *R* for each Δ*γ*.

**Figure 8:**
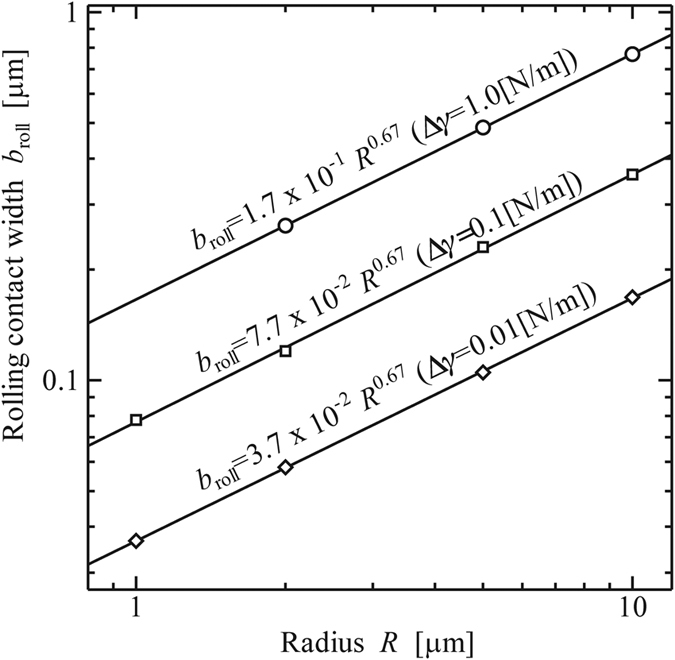
Width of contact area at the beginning of rolling, *b*_roll_ [μm]. (Material: polystyrene, *E* = 3.8 [GPa], *ν* = 0.34).

Figure 8 shows the relation between *b*_roll_ and *R* for Δ*γ* = 0.01, 0.1, and 1.0 [N/m]. The data shows that *b*_roll_ is nearly proportional to *R*^1/3^ for each Δ*γ*.

**Figure 9:**
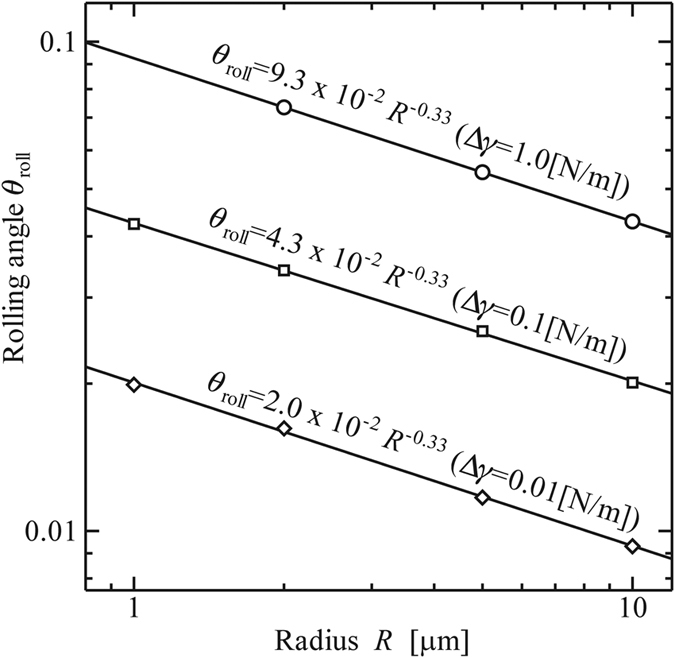
Rotational angle at the beginning of rolling, *θ*_roll_ [rad]. (Material: polystyrene, *E* = 3.8 [GPa], *ν* = 0.34).

Figure 9 shows the relation between *θ*_roll_ and *R* for Δ*γ* = 0.01, 0.1, and 1.0 [N/m]. The data shows that *θ*_roll_ is nearly proportional to *R*^−1/3^ for each Δ*γ*.

**Figure 10:**
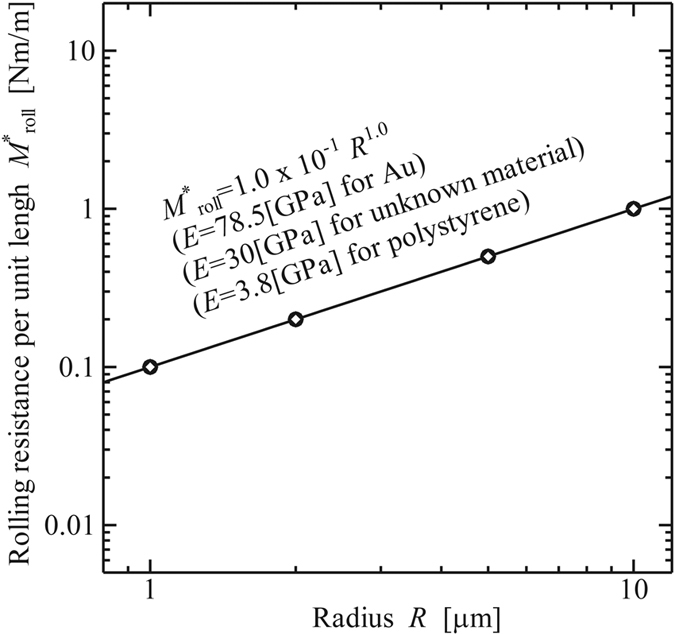
Rolling resistance per unit length at the beginning of rolling,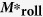 [10^−6^ Nm/m]. (Work of Adhesion: Δ*γ* = 0.1 [N/m]).

**Figure 11:**
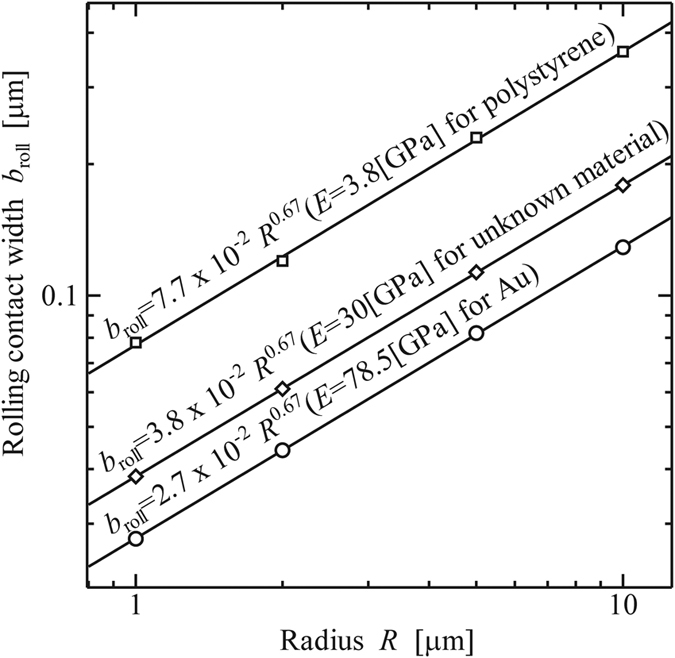
Width of contact area at the beginning of rolling, *b*_roll_ [μm]. (Work of Adhesion: Δ*γ* = 0.1 [N/m]).

**Figure 12:**
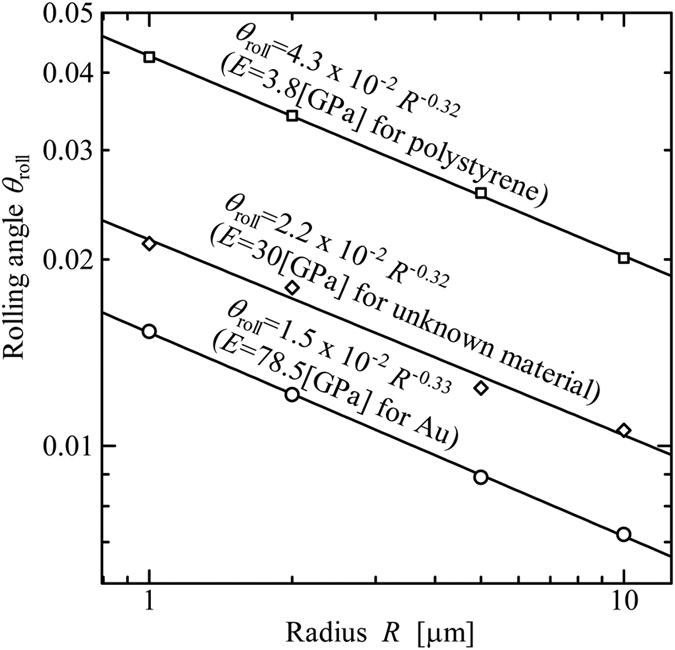
Rotational angle at the beginning of rolling *θ*_roll_ [rad]. (Work of Adhesion: Δ*γ* = 0.1 [N/m]).

Furthermore, to investigate the dependency of Young’s modulus for the normalized formulas, we also calculated 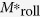, *b*_roll_, and *θ*_roll_, for various radii *R* and Δ*γ* = 0.1[N/m] for Au, as shown in Figs 10, 11 and 12, respectively.

The calculation results surprisingly shows that the value of 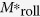 is independent of Young’s modulus although values of *b*_roll_, and *θ*_roll_ do depend on Young’s modulus.

The general expressions of 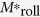, *b*_roll_, and *θ*_roll_ can be written in terms of *R*, Δ*γ*, and E*:


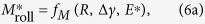



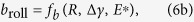



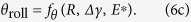


**Figure 13:**
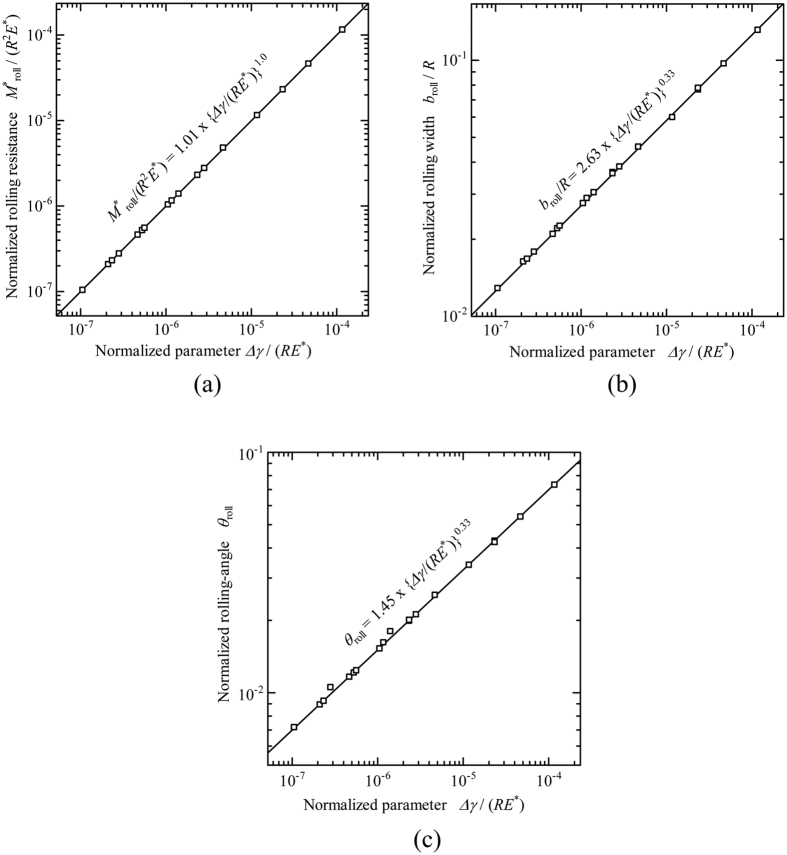
Relations between normalized and dimensionless values ((a) *M*_roll_/*R*^2^E*, (b) *b*_roll_/*R*, and (c) *θ*_roll_) and the normalized variable, Δ*γ*/RE*, plotted in logarithmic scales.

Using the ∏-theorem in dimensional analysis, we obtain the general dimensionless functions:


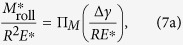



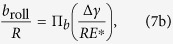



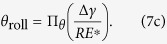


where ∏_*M*_, ∏_*b*_, and ∏_*θ*_, are normalized dimensionless functions. To extract approximate explicit functional forms of ∏_*M*_, ∏_*b*_, and ∏_*θ*_, FEM simulation results are plotted in logarithmic scales as shown in Fig. 13. We have confirmed that 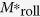/*R*^2^E*, *b*_roll_/*R*, and *θ*_roll_ are linearly related to Δ*γ*/RE* in logarithmic scales, which can be easily fitted with the functional form *c* (Δ*γ*/RE*)^*n*^ (*c* and *n*: are *c*onsta*n*ts). By using least-square method, we can now extract the constants, *c* and *n*, for each dimensionless function at the maximum coefficient of determination (*R*_det_) as follows:


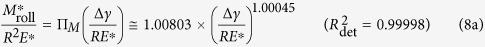



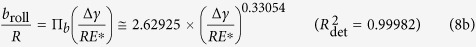



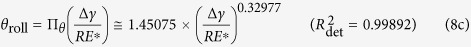


We have found out that the power indices of Δ*γ*/RE* for *M*_roll_/*R*^2^E*, *b*_roll_/*R*, and *θ*_roll_ in Eqs (8a), (8b), and (8c) are very close to 1, 1/3, and 1/3, respectively. We thus re-write Eq. (8) using the following functions with best fitting:


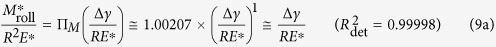



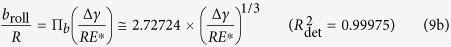



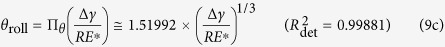


with all the coefficients of determination fairly close to 1. This is similar to Kalker’s earlier work^17^ where *b*_initial_/*R* was found to be proportional to the 1/3 power of Δ*γ*/RE* for the pull-off problem. These equations are formulas of rolling resistance in the normalized and dimensionless form for a cylindrical elastic object per unit length in adhesional contact.

Within the explored parameter space in this study, we obtain


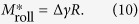


Surprisingly, the rolling resistance is determined only by ‘*work of adhesion times cylindrical radius*’ and independent of Young’s modulus (E*). According to Kalker’s theory^17^, the pull-off force *F*_pull-off_ per unit length to detach a cylindrical object from the plate should be (3/2)(πΔ*γ*^2^RE*/2)^1/3^, while the value of *a*_pull-off_, one half of *b*_pull-off_, should be (2Δ*γR*^2^/πE*)^1/3^. Thus we have *F*_pull-off_ *a*_pull-off_ = (3/2)Δ*γR*, also independent of Young’s modulus.

From the results of dimensional analysis, the solution of the rolling problem only depends on one single dimensionless variable, Δ*γ*/RE*. The parametric study covered the variable range for Δ*γ*/RE* across 3 orders of magnitude, i.e., from 1.05 × 10^−7^ to 1.16 × 10^−4^. Within this range, the normalized dimensionless functions we extracted provide us with a quantitative understanding about the rolling-resistance of a micro-cylinder.

### An asymptotic linear elastic fracture mechanics solution

In this section, we compare the full-field FEM results with an asymptotic solution based on the linear elastic fracture mechanics (LEFM) approach.

**Figure 14:**
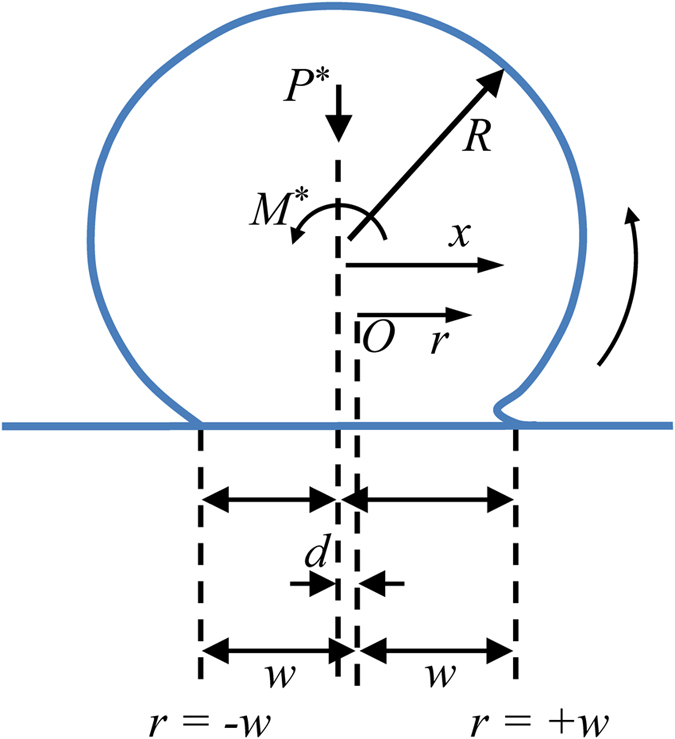
Schematic of a cylinder rolling on a flat plate counterclockwise. Here, the cylinder is deformable and the flat plate is rigid. Adapted from ref. 11.

As illustrated in Fig. 14 and following the asymptotic LEFM approach described in the literature^11^,^13^, the stress distribution on the interface can be analytically estimated as:


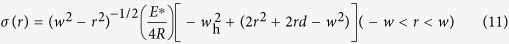


where *w*_h_ is the Hertzian, non-adhesive half width for a contact under the same vertical load, P*, and the center of the roller is *r* = −*d*. Note that *w* in Fig. 14 and the following discussion corresponds to *b*/2 denoted in earlier sections. Consequently, the moment per unit micro-cylinder length M* can be evaluated by integrating the stress distribution as:


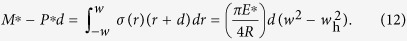


The corresponding stress intensity factors at *r* = ±*w* are


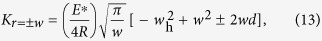


and the energy release rates at *r* = ±*w* are


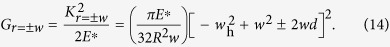


The net change in energy release rate between *r* = +*w* and *r* = −*w* can now be given as


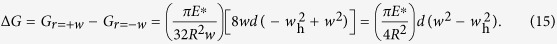


With Eq. (12) and Eq. (15), and setting Δ*G* = Δ*γ* at the incipient rolling we have


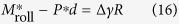


For the case with P* = 0 in Fig. 14, we obtain 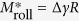, which is the same as we obtained earlier using full-field FEM analysis in Eqs (9a) and (10). In this case, the critical moment of initial rolling identified using the asymptotic LEFM approach agrees well with that obtained through FEM simulations.

For incipient (initial) rolling, the stress at the leading edge (*r* = −*w*) will be finite, i.e. *K*_*r*=−*w*_ = 0, then we have


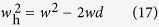


at the point of initial rolling. The change in mode I plane strain energy release rate in Eq. (15) can be rewritten as


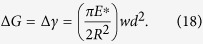


Note that when P* = 0 in Fig. 14, we have *w*_h_ = 0, and thus *w* = 2*d* after applying Eq. (17). Since the contact width *b* = 2*w* by definition, manipulating Eq. (18) gives


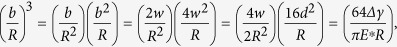


and thus at the initial rolling,


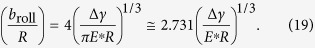


This again agrees well with 2.727, the coefficient we obtained in Eq. (9b) using FEM simulations. We can see an excellent agreement in contact width at the initial rolling. And at this point, *d* in Fig. 14 is now


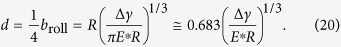


We further evaluate the initial rolling angle. For the definition of the rotational angle *θ* = *d*/*R*, the rolling angle can be obtained as


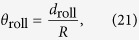


where*d*_roll_ = *d*′_roll_ + *d*_offset_; *d*′_roll_is the displacement given by Eq. (20) resulted from 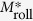 with the contact width *b*_roll_, and *d*_offset_ is the offset displacement due to the changing of contact width from *b*_initial_ to *b*_roll_ assuming the leading edge does not move. *d*_offset_ can be calculated from Eqs (1) and (19) as:


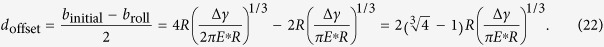


As a result, we obtain the rolling angle as the sum of two terms:


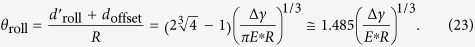


This result again agrees well with the full-field FEM solution given by Eq. (9c).

## Discussion and Concluding Remarks

As an example for a first order comparison with our results, Peri and Cetinkaya^10^ experimentally evaluated the value of rolling resistance moment of a micro-sphere. They described the value as *M*_roll_ = 6*π* Δ*γ ξ r* where*M*_roll_is the critical rolling resistance moment, Δ*γ* is the work of adhesion, *ξ* is the shift displacement in oscillation, *r* is the radius of the sphere. In the experiment of a polystyrene latex micro-sphere (with 21.4 μm in radius) on silicon, *ξ* was 29.96 nm, the contact radius of the micro-sphere and the silicon substrate *a* was 231 nm and contact area was 0.168 [μm^2^]; consequently *M*_roll_ would be *M*_roll_ = 6*π*Δ*γ* (29.96 [nm]) (21.4 [μm]) ≈ 12Δ*γ* × 10^−12^ [Nm] (Δ*γ* in [N/m]). Using the present theory for a micro-cylinder, we have 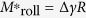 which corresponds to *M*_roll_ = Δ*γ R a* if we count only the contribution within a length *a* in the micro-cylinder. Therefore for a polystyrene latex micro-cylinder (with 21.4 μm in radius) on silicon, we have *M*_roll_ = Δ*γ* (21.4 [μm])(231 [nm]) ≈ 5Δ*γ* × 10^−12^ [Nm] (Δ*γ* in [N/m]). Note that the reduced modulus E* = 3.04 GPa for this case, and the estimated contact radius at incipient rolling is 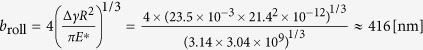, and the contact area is *b*_roll_ × *a* = 416 [nm] × 231 [nm] = 0.096 [μm^2^], which is comparable to the contact area for the micro-sphere. The predicted critical rolling moments are found to be on the same order of magnitude for a micro-cylinder and a micro-sphere, when the contact areas for these two cases are similar.

In summary, we obtained closed-form expressions describing the critical rolling resistance of an isotropically-elastic cylindrical micro-object in adhesional contact with a rigid surface in normalized and dimensionless forms. In order to systematically evaluate the critical conditions of the rolling resistance, we established the procedure using finite element method, taking into account the full field stress, strain, and strain energy distribution. By considering the energy balance of the system, we confirmed the existence of the contact area that provides the equilibrium state of the model. We calculated the rolling resistance with different cylinder radii, works of adhesion, and Young’s moduli in adhesional contact with a frictionless rigid surface, and numerically established the critical conditions of rolling resistance in closed-form dimensionless functions. The critical rolling moment was found to be determined only by *work of adhesion times cylindrical radius* and independent of Young’s modulus, while the initial rolling contact area and initial rolling angle still depend on Young’s modulus. In addition, building upon a linear elastic fracture mechanics based approach, exact asymptotic analytical solutions were obtained for the critical rolling moment, initial contact area and initial rolling angle; these asymptotic solutions compare very well with the full-field finite element results. In future work, we will study the rolling resistance for other geometries, and analyze the influence of the friction parallel to the substrate on the rolling resistance.

## Additional Information

**How to cite this article**: Saito, S. *et al.* Rolling behavior of a micro-cylinder in adhesional contact. *Sci. Rep.* **6**, 34063; doi: 10.1038/srep34063 (2016).
